# Metagenomes and Metatranscriptomes of Activated Sludge from a Sewage Plant, with or without Aerobic Granule Enrichment

**DOI:** 10.1128/genomeA.00372-17

**Published:** 2017-05-25

**Authors:** Julien Crovadore, Vice Soljan, Gautier Calmin, Romain Chablais, Bastien Cochard, François Lefort

**Affiliations:** a Plants and Pathogens Group, Institute Land Nature and Environment, hepia, HES-SO University of Applied Sciences and Arts Western Switzerland, Jussy, Geneva, Switzerland; b Puratis Sàrl, EPFL Innovation Park, Lausanne, Vaud, Switzerland; c Faculty of Engineering and Architecture, HES-SO University of Applied Sciences and Arts Western Switzerland, Delémont, Jura, Switzerland

## Abstract

We report here the metagenomes and metatranscriptomes of activated sludge bioreactors, enriched or not enriched with aerobic granules, at an initial state and after 1 month of incubation. Data showed that the added granular biomass expressed higher levels of expression of genes involved in ammonia elimination.

## GENOME ANNOUNCEMENT

The anaerobic digestion is commonly used for reducing sludge solids in wastewater treatment and can be improved by the wet oxidation process (WOX), which aims to convert carbon into methane through oxidation of organic compounds. WOX effluents are, however, rich in ammonia, the removal of which is necessary for maintaining the activity of methanogens. We therefore tested here the potential of aerobic granules to remove ammonia from WOX effluents. Two aerated glass bioreactors were inoculated with 5 liters of activated sludge (AS), at a starting concentration of 5 g/liter of mixed liquor suspended solids (MLSS). One bioreactor (B1) was inoculated with an AS from wet oxidation effluents of a wastewater treatment plant. The second bioreactor (B2) was inoculated with the same AS, added to 200 ml of granular biomass (aerobic granules [AG]). After the addition of 1 liter of a mixture made of wet oxidation effluent (ammonia concentration 400 mg/liter), leachate, and water, the granulation process of the AS was followed in both bioreactors for 1 month, with a four-step cycle (substrate addition, aeration, sedimentation and water decantation), repeated every 3 days and pH values maintained between 6 and 8 throughout the experiment. The final concentration of ammonia was between 250 and 300 mg/liter. Samples were taken at the start of the incubation and after 1 month: initial activated sludge (IAS), initial activated sludge + aerobic granules (IAS + AG), final activated sludge (FAS), and final activated sludge + aerobic granules (FAS + AG). They were collected in 50-ml Falcon RNase and DNase free tubes and total nucleic acids were extracted with the PowerMax soil DNA isolation kit (MO-BIO Laboratories, Inc., CA, USA) while RNAs were purified with Powersoil total RNA isolation kit (MO-BIO Laboratories). DNA traces were removed from RNA samples with the amplification grade DNase I kit (Sigma-Aldrich, Inc., MO, USA). Prior to the construction of metatranscriptomic libraries, prokaryotes, and eukaryotes rRNAs were depleted with the MICROBExpress kit (Ambion, Thermo Fisher Scientific, Inc., Switzerland). Metagenomic libraries were built using the Illumina TruSeq nano DNA preparation kit (Illumina, CA, USA) and cDNA libraries with the Illumina TruSeq stranded mRNA sample preparation kit. Finally, all libraries were multiplexed and sequenced in one single run (2 × 100 bp) in an Illumina Hiseq2000 sequencer. The resulting sequencing depths of metagenomes ranged from 4.064 to 7.599 Gbp, and between 4.001 and 5.833 Gbp for the metatranscriptomes. After quality control was performed with FastQC (http://www.bioinformatics.babraham.ac.uk/projects/fastqc) and quality trimming with Trimmomatic version 0.27 (1), metagenomes analyses, for taxonomy and identification of genes of the nitrogen metabolism, were performed in the MG-RAST pipeline (2). The comparison of the annotated species and of 7 genes of the nitrogen metabolism between both conditions and times of sampling showed that bacterial communities increased the expression level of the nitrogen metabolism and that aerobic granules contributed to the reduction of ammonia more than in the control without aerobic granules. Enriching in granular biomass could therefore help to reduce the costs of ammonia removal in sewage plants and might result in reduction of chemicals and energy consumption.

### Accession number(s).

Metagenome and metatranscriptome raw sequencing data sets have been made public through the Sequence Read Archive (SRA) (3) of the National Center for Biotechnology Information under the SRA accession numbers given in Table 1. They have also been deposited at the MG-RAST database (accessible at http://metagenomics.anl.gov/).

**TABLE 1:** SRA accession numbers

Sample name	SRA accession no.
IAS_DNA_	SRP050327
FAS_DNA_	SRP051963
IAS + AG_DNA_	SRP052005
FAS + AG_DNA_	SRP052006
IAS_cDNA_	SRP052007
FAS_cDNA_	SRP052008
IAS + AG_DNA_	SRP052009
FAS + AG_DNA_	SRP052010
